# Survival and growth of *Stenotrophomonas maltophilia* in free-living amoebae (FLA) and bacterial virulence properties

**DOI:** 10.1371/journal.pone.0192308

**Published:** 2018-02-05

**Authors:** Elodie Denet, Valentin Vasselon, Béatrice Burdin, Sylvie Nazaret, Sabine Favre-Bonté

**Affiliations:** 1 Université Lyon 1, UMR CNRS 5557/UMR INRA 1418 Ecologie Microbienne, Villeurbanne, France; 2 Université Lyon 1, Centre Technologique des Microstructures, Villeurbanne, France; University of Padova, Medical School, ITALY

## Abstract

*Stenotrophomonas maltophilia* is found ubiquitously in the environment and is an important emerging nosocomial pathogen. *S*. *maltophilia* has been recently described as an Amoebae-Resistant Bacteria (ARB) that exists as part of the microbiome of various free-living amoebae (FLA) from waters. Co-culture approaches with *Vermamoeba vermiformis* demonstrated the ability of this bacterium to resist amoebal digestion. In the present study, we assessed the survival and growth of six environmental and one clinical *S*. *maltophilia* strains within two amoebal species: *Acanthamoeba castellanii* and *Willaertia magna*. We also evaluated bacterial virulence properties using the social amoeba *Dictyostelium discoideum*. A co-culture approach was carried out over 96 hours and the abundance of *S*. *maltophilia* cells was measured using quantitative PCR and culture approach. The presence of bacteria inside the amoeba was confirmed using confocal microscopy. Our results showed that some *S*. *maltophilia* strains were able to multiply within both amoebae and exhibited multiplication rates up to 17.5 and 1166 for *A*. *castellanii* and *W*. *magna*, respectively. In contrast, some strains were unable to multiply in either amoeba. Out of the six environmental *S*. *maltophilia* strains tested, one was found to be virulent. Surprisingly, this strain previously isolated from a soil amoeba, *Micriamoeba*, was unable to infect both amoebal species tested. We further performed an assay with a mutant strain of *S*. *maltophilia* BurA1 lacking the efflux pump *eby*CAB gene and found the mutant to be more virulent and more efficient for intra-amoebal multiplication. Overall, the results obtained strongly indicated that free-living amoebae could be an important ecological niche for *S*. *maltophilia*.

## Introduction

*Stenotrophomonas maltophilia* is a non-fermentative Gram-negative bacterium occurring ubiquitously in various natural and anthropogenic environments [1]. The presence of S. *maltophilia* has been reported in various water sources such as rivers [2], petroleum reservoir waste water in Iran [3], high altitude lakes, as well as in sediment [4] and deep-sea invertebrates [5]. This species also occurs in various soil types all around the world [6,7] where it is a frequent colonizer of the rhizosphere [8,9]. This bacterium shows plant-growth promoting activity as well as antagonistic properties against bacterial and fungal plant pathogens due to its production of phytohormones [10] and chitinolytic activities [11]. It can also degrade a variety of xenobiotics [12,13] and hydrocarbons [14] with a significant role in bioremediation of polluted sites [15]. This bacterium was also found associated with the gut of a bark beetle where it could be implicated in the oxidation, fermentation, and hydrolysis of cellulose and lignin derived aromatic products [16]. Recently, we showed that *S*. *maltophilia* is also part of the microbiome of several free-living amoebal genera from soils collected in Burkina Faso and Vietnam [17]. However, its role in the context of amoebal interactions is poorly known.

*S*. *maltophilia* is also described as an important nosocomial pathogen responsible for severe infections such as bacteremia, endocarditis, pneumonia, and urinary tract infections among immunocompromised patients [18]. It can also cause infections in animals such as respiratory infections with chronic coughing in horses, canines, and bovines [19–21]. One of the major features of *S*. *maltophilia* is the presence of numerous antibiotic resistance coding genes and efflux pump operons that confer frequent Multi-Drug Resistant (MDR) phenotypes among both clinical and environmental isolates [7]. Its genome is also characterized by the presence of several genes involved in virulence such as hemolysin, protease, phospholipase genes, and the *smf*1-operon which permits biofilm formation [22]. While all *S*. *maltophilia* strains have genes conferring virulence, not all of them are virulent. Indeed, the virulence of 59 strains of *S*. *maltophilia* was tested with *Dictyostelium discoideum* amoebal model and it was observed that environmental isolates were less virulent than clinical strains. Furthermore, this study showed that the virulence differed when the strains were tested with *D*. *discoideum* or *Acanthamoeba castellanii* [23].

Most work studying the interactions between amoebae and bacteria focused on *Acanthamoeba sp*. and *L*. *pneumophila* [24,25]. Only two reports from the literature mentioned a co-culture approach between various amoebal species and *S*. *maltophilia* and showed that *S*. *maltophilia* was able to resist amoebal digestion and even to grow inside the host [26,27]. As these studies focused on three strains of *S*. *maltophilia* (patient’s blood culture, hospital water and intra-amoebal bacteria) the conclusion might not be representative of the interaction between *S*. *maltophilia* and free-living amoebae.

In this context, the aim of the present study was to determine the survival and growth of various environmental strains of *S*. *maltophilia* within two common environmental amoebal species *A*. *castellanii* and *Willaertia magna* and compare their virulence properties. To achieve this purpose, a co-culture approach with these two amoebae was carried out and the abundance of *S*. *maltophilia* cells was measured over time using real time quantitative PCR. In parallel, we confirmed the presence of bacteria inside amoeba using confocal microscopy and the viability and multiplication of intramoebal bacteria using a culture approach. Virulence was assessed using the social amoeba, *Dictyostelium discoideum*.

## Materials and methods

### Bacterial strains and growth conditions

Five environmental strains of *S*. *maltophilia* from our team’s collection were used in this study (Table 1). Two strains (BurA1 and BurE1) were isolated from bulk soil samples collected in sorghum fields in Burkina Faso [7], one strain (PierC1) was isolated from an agricultural soil contaminated with heavy metals, antibiotics, and xenobiotics in the Pierrelaye plain (France). Two strains (MEEB-Am6.1 and MEEB-Am6.2) were isolated by a culturable method from two different amoebal genera i.e. *Micriamoeba* and *Tetramitus* and two different soils, Vietnam and Burkina Faso, respectively [17]. Two reference strains of *S*. *maltophilia* were added: the clinical reference strain K279a [28] and the environmental reference strain R551-3 [29].

**Table 1:** Strains and plasmids used in this study.

Strains or plasmids	Genotype or properties	References
*S*. *maltophilia* BurA1	Wild type, soil strain	[7]
*S*. *maltophilia* BurE1		
*S*. *maltophilia PierC1*		
*S*. *maltophilia* MEEB-Am6.1	Intra-amoebal bacteria	[17]
*S*. *maltophilia* MEEB-Am6.2		
*S*. *maltophilia* K279a	Clinical reference strain	[28]
*S*. *maltophilia* R551.3	Environmental reference strain	[29]
BurA1Δ*eby*CAB	*S*. *maltophilia* BurA1 mutant of *eby*CAB operon, Δ*eby*CAB	This study
*Escherichia coli DH5*a	F^-^ Ф80d*lacZ*ΔM15 Δ(*lacZYA-argF*)*U169 deoR recA1 endA1 hsdR17* (rk- mk+) *phoA supE44*, *thi-1 gyrA96 relA1*λ-	Invitrogen
*E*. *coli* S17-1	λpir-positive mating strain	In the laboratory
plasmid pGEMT-easy	Amp^r^, *lac*Z	Promega
plasmid pEX18Tc	*sacB*, *oriT*, Tc^r^	Promega
plasmid pGEM-UD	pGEMT-easy containing the upstream and downstream regions of *eby*CAB operon	This study
plasmid pEX18-UD	pEX18Tc containing the upstream and downstream regions of *eby*CAB operon	This study

*Pseudomonas aeruginosa* PT5 [30] and *Klebsiella pneumoniae* KpGe (Lima *et al*., 2017; unpublished work) were used as reference strains in virulence assays. One day before each experiment, bacteria were sub-cultured in Luria-Bertani (LB) broth at 28°C, and shaken at 180 r.p.m. overnight.

A BurA1 mutant lacking the previously described efflux pump *eby*CAB gene [7] was also used in this study. To construct the BurA1Δ*eby*CAB mutant, two regions of a 1 kb long fragment located upstream and downstream (UD) from the *eby*CAB operon were amplified from the genome of *S*. *maltophilia* BurA1 using primers upBurA1-F/upBurA1-R and dwBurA1-F/dwBurA1-R (Table 1). The 952-bp and 1018-bp PCR products were subsequently hybridized using complementary regions introduced in primers. The UD fragment obtained was cloned into the vector pGEM-T (Promega) yielding plasmid pGEM-UD. The plasmid was introduced into *Escherichia coli* DH5α. The plasmid pGEM-UD was then digested by *Eco*RI in order to release the UD fragment, which was next cloned into plasmid pEx18-Tc. The plasmid pEx18-UD was introduced into *E*. *coli* S17-1 by transformation and mobilized into *S*. *maltophilia* BurA1 *via* conjugation. Transconjugants carrying deleted *eby*CAB in the chromosome after double-crossover homologous recombination were obtained by a two-step selection on LB agar containing tetracycline (10 μg/ml) / imipenem (32 μg/mL) and then on LB agar containing 10% (wt/vol) sucrose, yielding the deletion mutants BurA1Δ*eby*CAB (S1 Fig). The correctness of mutant was confirmed by colony PCR.

### Amoebal strains and growth conditions

Two axenic free-living amoebae were used to evaluate survival and growth of *S*. *maltophilia* strains: *Acanthamoeba castellanii* L6a and *Willaertia magna* C2c (kindly provided by Michel Pélandakis, Microbiology, adaptation, pathogeny laboratory, University Lyon 1). They were grown in proteose peptone-yeast-glucose (PYG90) medium supplemented with fetal calf serum (10%) as monolayers in 75 cm^2^ tissue culture flasks at 28°C.

The axenic *D*. *discoideum* strain AX2 (kindly provided by Anne Vianney, CIRI, University Lyon 1) was used for virulence assays. Amoebal cells were grown in cell culture flasks in HL5 Medium [30] at 22.5°C.

### Co-cultures of *S*. *maltophilia* and free-living amoebae

For co-culture experiments, amoebae were harvested by tapping flasks and adherent trophozoites were washed twice with Page’s Amoeba Saline buffer (PAS) (2.5 mM KH_2_PO_4_, 4 mM MgSO_4_, 0.5 mM CaCl_2_, 2.5 mM, NA_2_HPO_4_, 0.05 mM (NH_4_)_2_FeII(SO_4_)_2_) by centrifugation (1000 x g, 10 min). The pellet containing amoebal cells was resuspended in PAS supplemented with glucose and yeast extract (in order to avoid encystement) and the final concentration of cells was adjusted to 1.1 x 10^5^ cells.mL^-1^. One milliliter of each trophozoite suspension was distributed to each well of a 24-well microplate. Microplates were incubated at 25°C for two hours to allow adhesion of amoebal cells. At the same time, *S*. *maltophilia* suspension from LB broth was diluted in PAS buffer at a concentration of 2 x 10^6^ cells.mL^-1^. Then, 100 μL of bacterial suspension was added to each well containing the amoebal cells (multiplicity of infection 2). Microplates were centrifuged at room temperature (1890 x g, 10 min) to enhance contact between bacteria and trophozoite then were incubated at 25°C for one hour. The PAS was removed, two washing steps in PAS were performed and PAS containing gentamycin (200 μg.mL^-1^) was added to kill extracellular bacteria by incubating microplates one hour at 25°C. The Minimal Inhibitory Concentration of each *S*. *maltophilia* strain was previously determined and revealed they are susceptible to 200 μg.mL^-1^ gentamycin. PAS containing gentamycin was removed, one washing step was performed and PAS was added and microplates ware incubated at 32°C for 96 hours. Samples were harvested at 0, 24, 48, 72, and 96 hours by scraping wells and cell suspensions were used for DNA extraction and quantitative PCR. At each time sampling was performed in triplicate. Co-culture experiments were performed independently to quantify intra-amoebal *S*. *maltophilia* by culture approach. The enumeration of intra-amoebal bacteria was realized in duplicate at each kinetic time (0, 24, 48, 72 and 96 hours) by scraping wells and lysing amoebae by pipetting during 2 min 30 sec with a 25G needle. Recovered *S*. *maltophilia* were serial diluted, spotted onto agar plate and enumerated the following day (given as colony-forming-unit per mL).

### Real-time quantitative PCR (qPCR)

Samples were taken at different post-infection times and total DNA was extracted using Wizard SV Genomic DNA Purification System (Promega, Charbonnières-les-Bains, France). Abundance of *S*. *maltophilia* inside amoebae was quantified in duplicate using qPCR with a set of mono-copy gene-specific primers: smeD3: 5’ -CCAAGAGCCTTTCCGTCAT- 3’ and smeD5: 5’-TCTCGGACTTCAGCGTGAC-3’ [32]. qPCR amplification was performed using CFX-96 Connect (Bio-Rad, Marnes-la-Coquette, France) in a 25 μl volume containing 10 μL of Eva Green PCR Mastermix (Bio-Rad, Marnes-la-Coquette, France), 20 pmol of each primer and 5 μL of DNA template. The amplification conditions were as follows: 98°C for 15 minutes, followed by 45 cycles of 98°C for 10 seconds, 63°C for 20 seconds and 72°C for 15 seconds. Fluorescence was measured at the end of each cycle at 72°C and a melting curve analysis (65–98°C) was performed at the end of the amplification procedure.

### Confocal microscopy

In order to visualize potential survival of *S*. *maltophilia* in amoebae, confocal microscopy was performed on co-cultures. After the incubation periods (0 to 96 hours), cells within the 24-well microplates were fixed with 4% paraformaldehyde (Electron Microscopy sciences, Hatfield) for 30 minutes. Cells were then washed twice with Phosphate Buffer Saline (PBS: 8 g.L^-1^ NaCl, 0.2 g.L^-1^ KCl, 1.44g NA_2_HPO_4_, 0.24 g.L^-1^ KH_2_PO_4_) and were permeabilized for 10 minutes with 0.1% Triton. Coverslips were incubated for one hour at room temperature in PBS in wet room with primary rat antisera directed against total proteins of *S*. *maltophilia* (Abcam, Cambridge, United Kingdom). After washing twice with PBS, coverslips were incubated in wet room for one hour with second anti-rat antibodies coupled to Alexa Fluor 488 (488- emission 505) (Abcam, Cambridge, United Kingdom) in PBS containing concanavalin A (Cayman Chemical company, Ann Arbor, Michigan) in order to label amoebae red. After three washings with PBS and one with deionized water, the coverslips were mounted onto glass slides using the mounting medium Mowiol (Sigma-Aldrich, MO). The observations were performed on a Zeiss confocal microscope LSM800 (Munich, Germany) using a x63 apochromatic objective (NA 1.4), 0.7 μm optical sections and photos were analyzed using Zen software for microscopy. All experiments were performed in triplicate.

### Virulence assays

*S*. *maltophilia* virulence was determined as previously described [31] using the social amoeba, *D*. *discoideum*. Strains of *P*. *aeruginosa* PT5 and *K*. *pneumoniae KpGe* were used as negative and positive controls, respectively, for each assay. From the overnight bacterial culture, the optical density (OD) at 600 nm was adjusted to 1.5 by dilution in LB. For co-cultures between bacteria and *D*. *discoideum*, Sm Agar (FORMEDIUM, Hustanton, United Kingdom) medium was used. One mL of each bacterial suspension was spread on Sm Agar and plates were allowed to dry for one hour to obtain a dry bacterial layer.

Meanwhile, cells of *D*. *discoideum* were washed twice in PAS buffer by centrifugation at 1000 g for 10 minutes. The amoebal suspension was adjusted to 2 x 10^6^ cells.mL^-1^ and diluted in series to reach a final concentration of 7812 cells.mL^-1^. Five μL of each serial dilution was spotted on the bacterial lawn. Plates were incubated at 22.5°C for five days and appearance of phagocytic plaques was checked at the end of the incubation time. This assay was performed in triplicate.

In order to interpret the results, we used the categories defined by Adamek *et al*. (2011) [23]: non virulent (less than 400 amoebae for lysis plaque formation), low-virulent (400–2500 amoebae for lysis plaque formation) and virulent (more than 2500 amoebae).

### Statistical analysis

Statistical analysis was carried out using non-parametric Kruskal-Wallis to determine statistical differences between groups and non-parametric Friedman test to determine statistical differences between kinetic times into one specific group.

## Results

### Internalization and intracellular growth of *S*. *maltophilia* strains in *A*. *castellanii* L6a

To specifically detect and quantify *S*. *maltophilia* cells inside amoeba, we targeted the *sme*D gene. Based on the results of previous genome sequencing, one copy of the *sme*D gene was considered to be equivalent to one cell [7].

**Fig 1:**
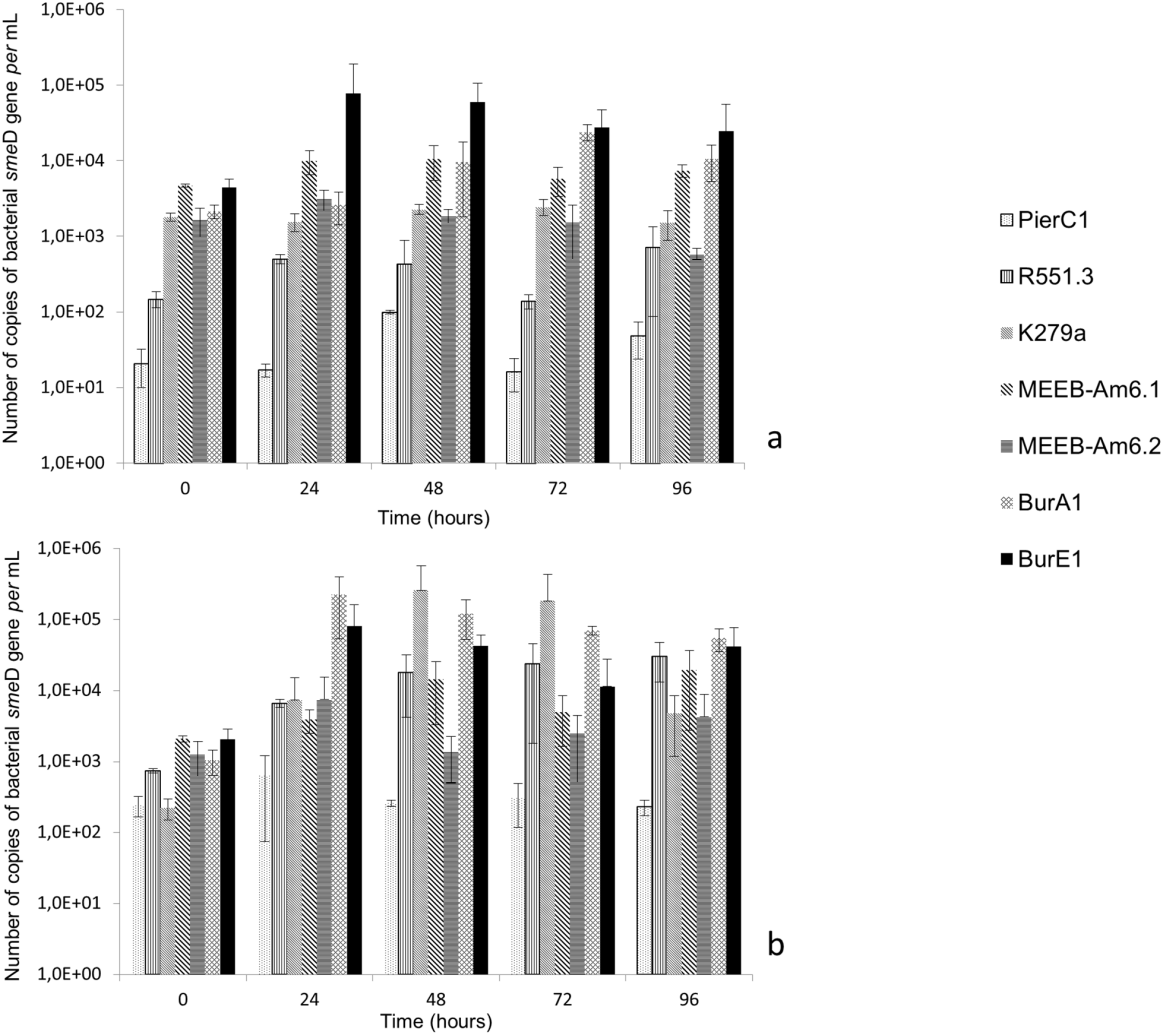
Growth of *S*. *maltophilia* strains expressed in number of copies of bacterial *sme*D gene *per* mL, in co-culture with amoeba. a) co-culture *with Acanthamoeba castellanii* L6a; b) co-culture with *Willaertia magna* C2c. Means +/- standard deviations from three independent experiments in duplicate are presented.

The beginning of the co-culture experiments (0 h) corresponded to the number of internalized cells. *A*. *castellanii* L6a had internalized about 3 x 10^3^ cells of *S*. *maltophilia* strains BurA1, BurE1, MEEB-Am6.1, MEEB-Am6.2 and K279a *per* mL. It internalized 2 x 10^1^ cells of PierC1 and 1.5 x 10^2^ cells of R551.3 *per* mL (Fig 1a). The difference between both groups of strains was statistically significant (p < 0.05).

**Fig 2:**
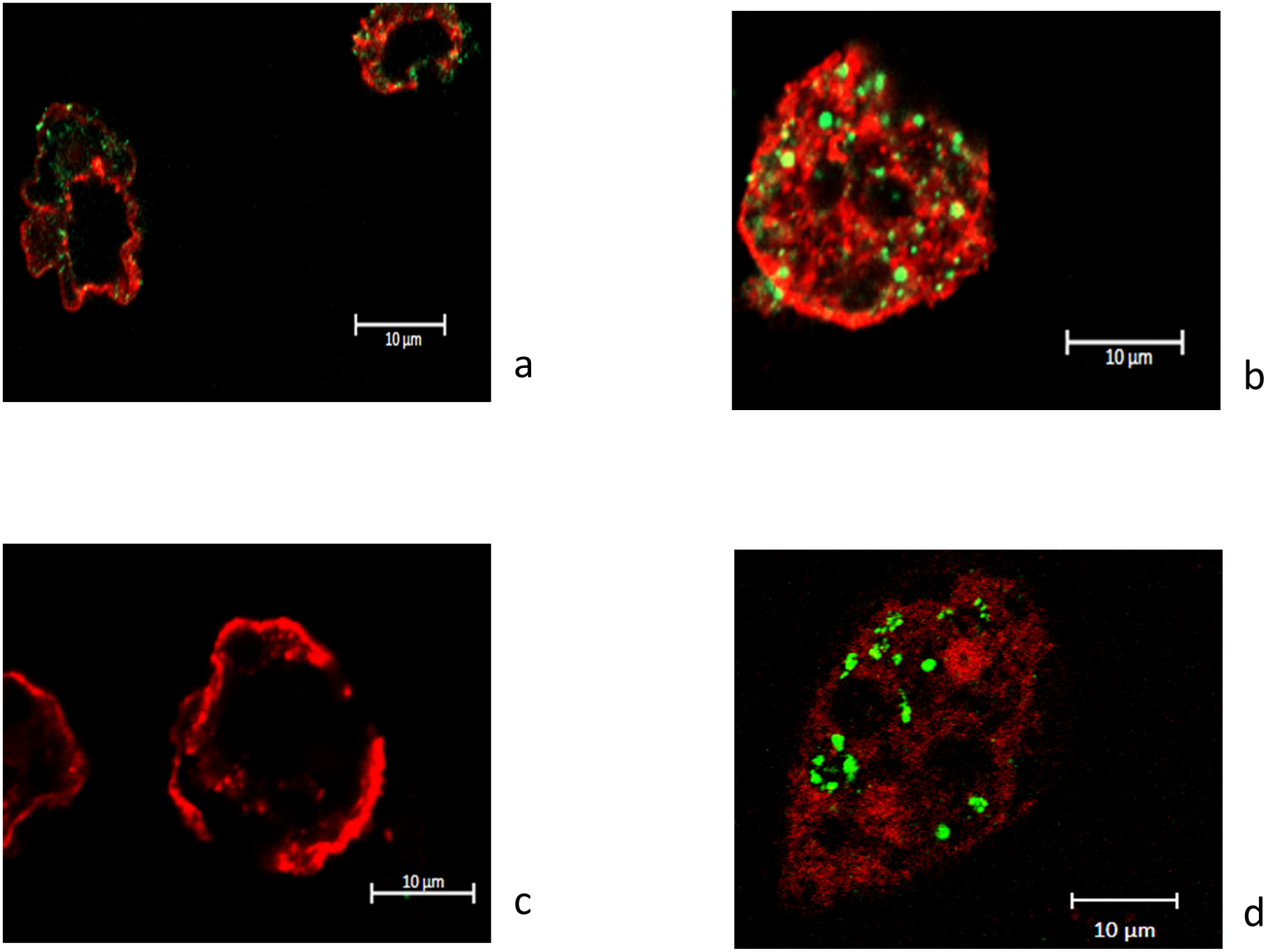
Fluorescent confocal microscopy images of *Acanthamoeba castellanii* L6a in co-culture with *S*. *maltophilia* strains. (a) after 48 hours with BurA1, (b) after 24 hours with BurE1, (c) after 24 hours with PierC1 and (d) after 48 hours with MEEB-Am6.2.

The number of *S*. *maltophilia* BurE1 inside *A*. *castellanii* increased by about 2 log after 24 hours of co-culture (p < 0.05), and the number of strain BurA1 increased by 1.5 log after 72 hours (p < 0.05). The number of *S*. *maltophilia* MEEB-Am6.1 and MEEB-Am6.2 remained stable during the entire course of the co-culture (p < 0.05). With the fluorescent confocal microscopy approach cells of strains BurA1, BurE1 and MEEB-Am6.2 were found in the cytoplasm of *A*. *castellanii* L6a (Fig 2a, 2b and 2d).

At the end of the experiment (96 hours), the number of *S*. *maltophilia* BurA1 and BurE1 was always greater than the number of cells at time 0. After 96 hours, cells of strains BurA1, BurE1, were still found inside the amoeba. Regarding the other strains, the number of cells remained relatively constant during the entire course of the experiment (Fig 1a). Using confocal microscopy cells of strains PierC1, MEEB-Am6.1, R551.3 and K279a were not visible in *A*. *castellanii* after 24 hours (Fig 2c) or later during the experiment.

### Internalization and intracellular growth of *S*. *maltophilia* strains in *W*. *magna* C2c

At the beginning of the co-culture experiments (0 h), *W*. *magna* C2c had internalized about 2 x 10^2^ cells of strains K279 and PierC1 *per* mL and about 7.5 x 10^2^ cells of strain R551.3 *per* mL whereas *W*. *magna* C2c had internalized about 1 x 10^3^ to 2 x 10^3^ cells of strains BurA1, BurE1, MEEB-Am6.1 and MEEB-Am6.2, *per* mL (Fig 1b). The difference between K279a and PierC1 strains, and the other strains was statistically significant (p < 0.05).

**Fig 3:**
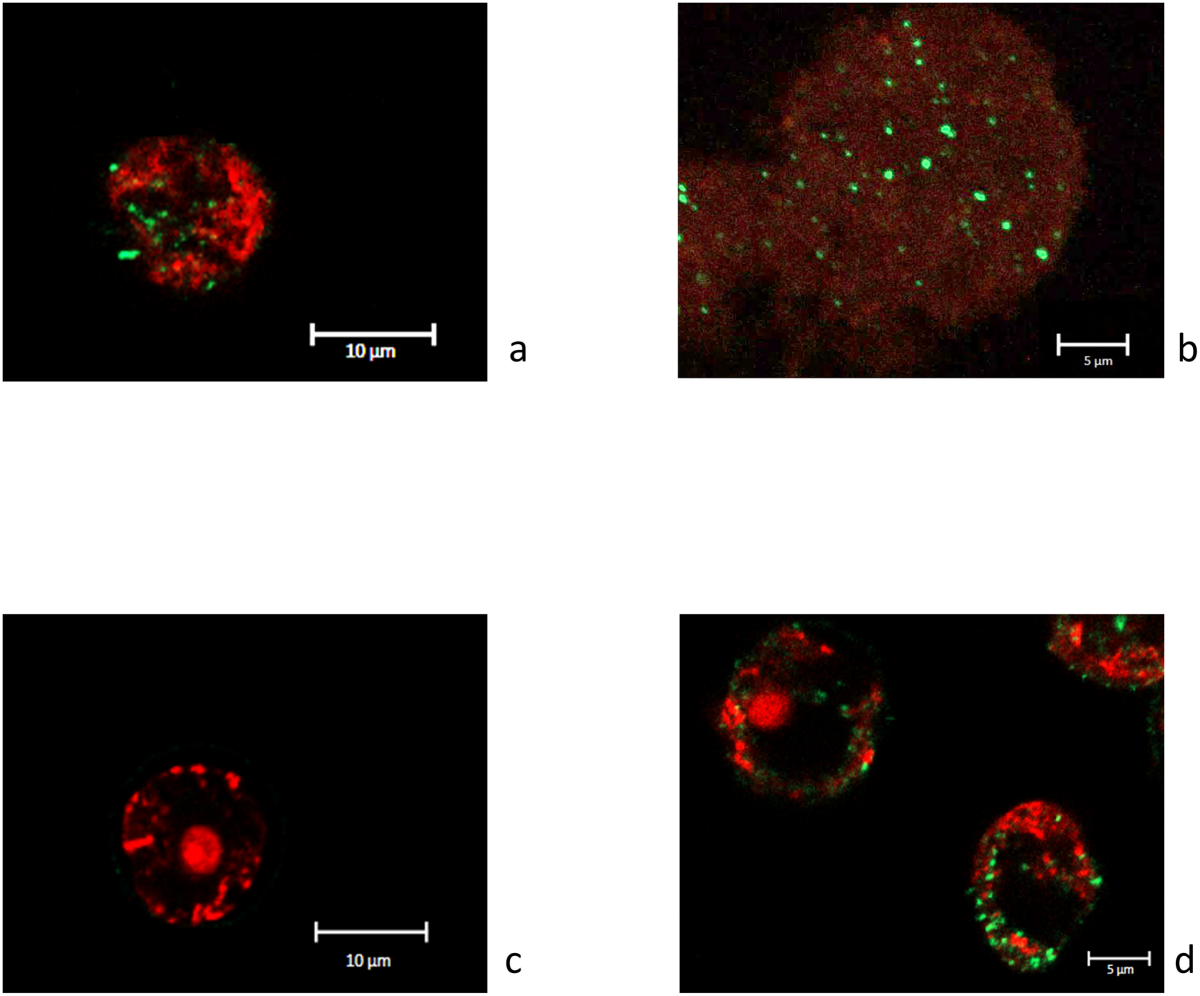
Fluorescent confocal microscopy images of *Willaertia magna* C2c in co-culture with *S*. *maltophilia* strains. (a) after 48 hours with BurA1, (b) after 72 hours with BurE1, (c) after 24 hours with PierC1 and after (d) 48 hours with K279a.

During the co-culture experiment with *W*. *magna* C2c, *S*. *maltophilia* BurA1, BurE1 and K279a replicated at the highest rates. After 24 hours of co-culture, the number of strains BurA1 and BurE1 increased by about 2.5 log and about 2 log respectively and, after 48 hours of co-incubation the number of strain K279a increased by about 3.5 log (p < 0.05). Fluorescent confocal microscopy experiments confirmed that cells of strains BurA1, BurE1 and K279a were inside the cytoplasm of *W*. *magna* C2c (Fig 3).

Two other strains of *S*. *maltophilia* were able to replicate to a lesser extent. After 24 hours of co-incubation, the number of strains MEEB-Am6.1 and MEEB-Am6.2 increased by about 0.5 log and 1 log respectively (p < 0.05). The number of *S*. *maltophilia* MEEB-Am6.1 remained stable during the entire course of the experiment whereas those of MEEB-Am6.2 increased by 2 log after 48 hours (p < 0.05). Cells of both strains were detected in the amoeba during the co-culture experiments as seen by confocal microscopy (Fig 3b).

*S*. *maltophilia* R551.3 presented a more gradual growth and the number of cells increased by about 1.5 log after 96 hours.

The number of strains PierC1 did not vary during the experiment and microscopy did not allow to detect bacteria in the amoeba regardless of the incubation length (Fig 3c).

### Viability of intra-amoebal *S*. *maltophilia*

**Fig 4:**
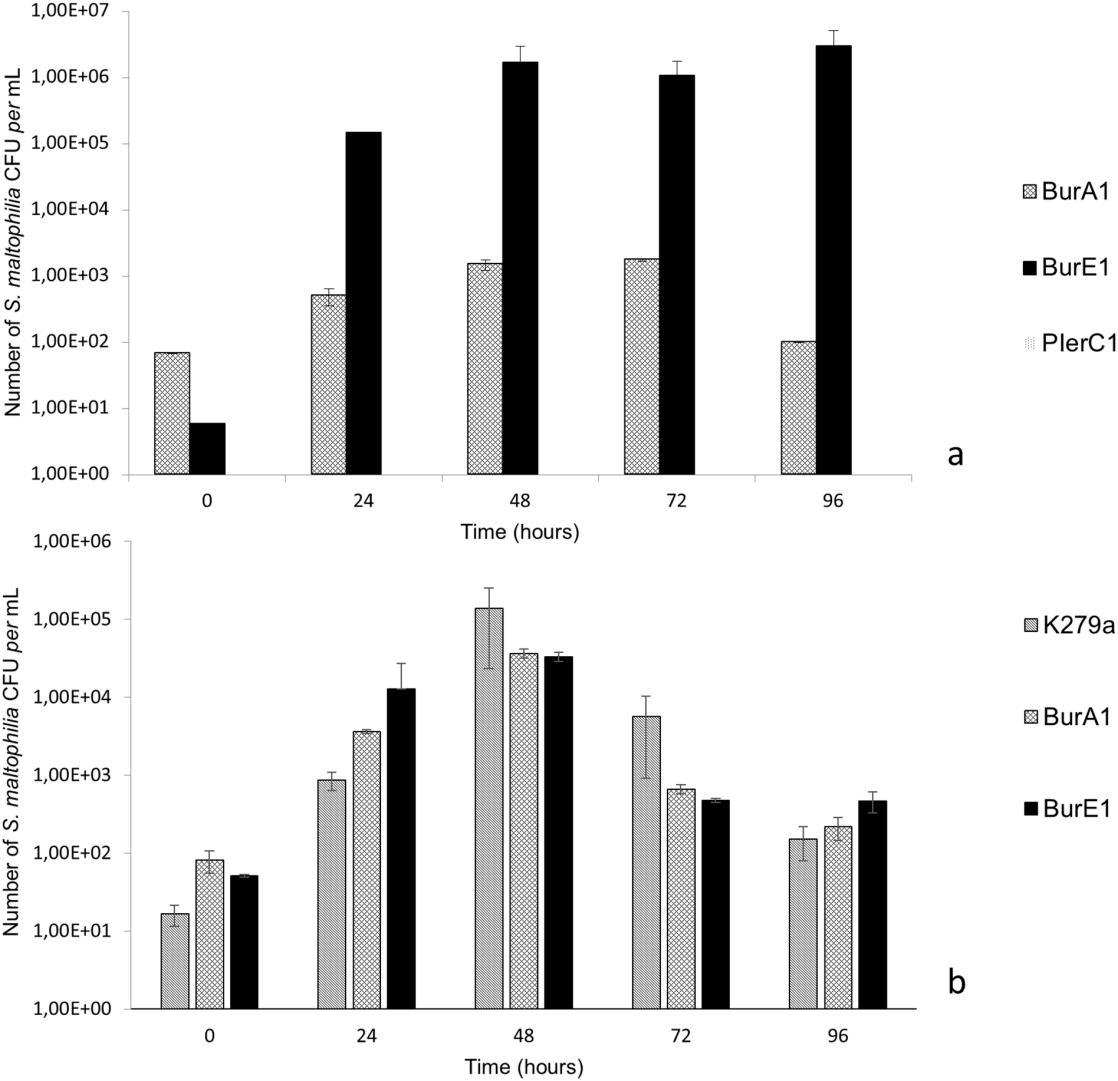
Growth of *S*. *maltophilia* strains in co-culture with amoeba, expressed in number of colony forming unit of *S*. *maltophilia per* mL. a) co-culture *with Acanthamoeba castellanii* L6a; b) co-culture with *Willaertia magna* C2c. Means +/- standard deviations from two independent experiments in triplicate are presented.

The ability of *S*. *maltophilia* strains to multiply inside the amoebae was performed using a culture approach. Representative strains among those showing multiplication properties by qPCR approach were co-cultivated with *A*. *castellanii* and *W*. *magna*. The number of CFU per mL of co-culture was determined after lysis of amoebae and plating intra-amoebal lysate on agar plates. The number of alive *S*. *maltophilia* BurE1, BurA1 and MEEB-Am6.2 strains in *A*. *castellanii* demonstrated increase by about 4–5 log, 1.5 log and 2 log respectively during the entire course of co-culture (Fig 4a). The PierC1 strain not able to replicate as quantified by qPCR approach was not detected using the culture approach.

In *W*. *magna* the number of *S*. *maltophilia* BurE1 and BurA1 increased by about 2.5 log, and K279a increased by 4 log during the entire course of co-culture (p < 0.05) (Fig 4b).

### Virulence of *S*. *maltophilia* strains

**Fig 5:**
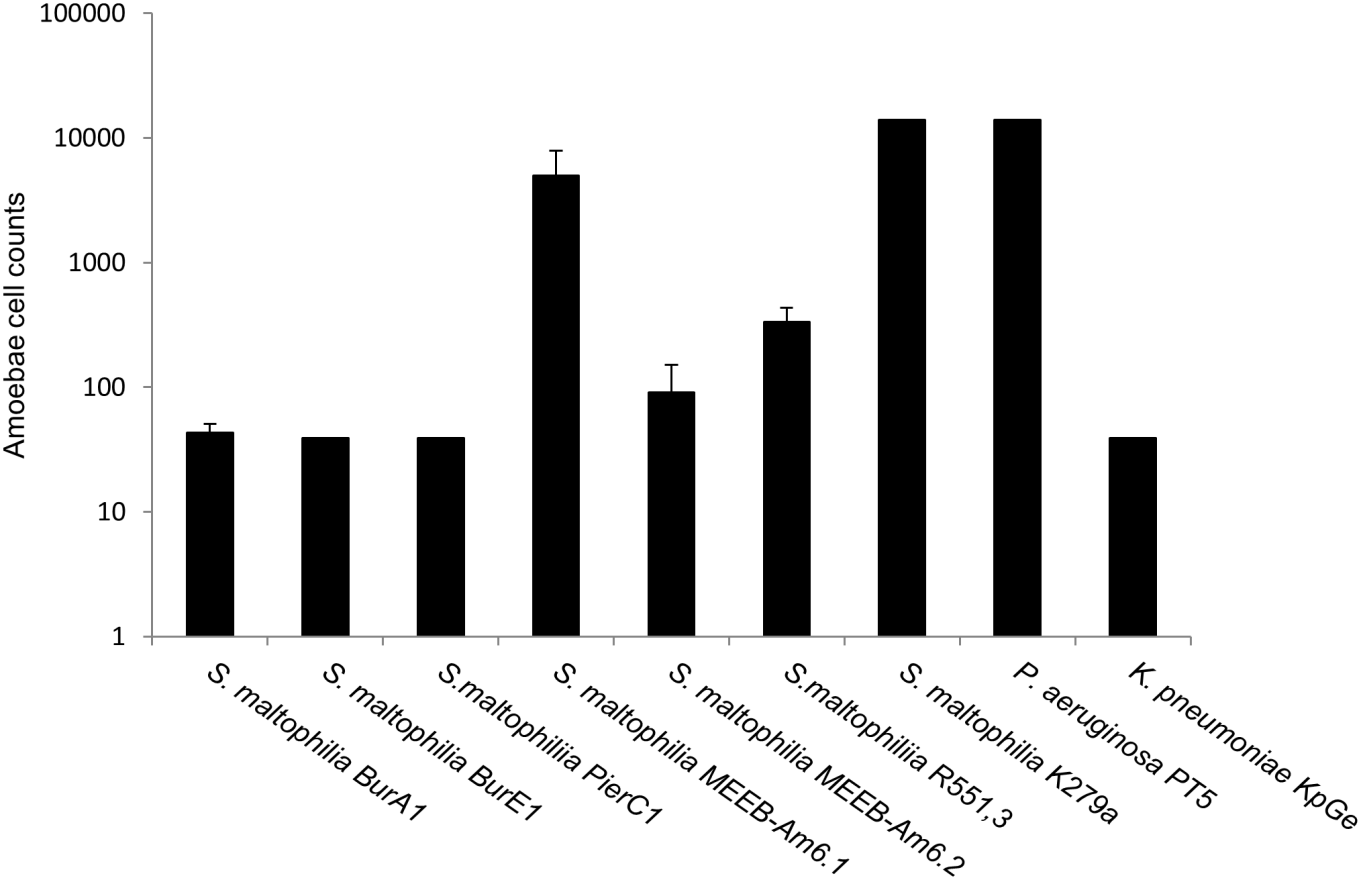
*D*. *discoideum* plate killing assay with seven *S*. *maltophilia* strains. Bars representing the number of amoebae necessary to form a lysis plaque on the bacterial lawn. *P*. *aeruginosa* PT5 and *K*. *pneumoniae* KpGe were used as negative and positive controls, respectively. Means +/- standard deviations from 3 independent experiments in triplicate are presented.

Fig 5 showed the virulence of the various *S*. *maltophilia* strains towards *D*. *discoideum*. *K*. *pneumoniae* KpGe and *P*. *aeruginosa PT5* were used as a positive control for a non-virulent strain and negative control of a virulent strain, respectively.

Five strains of *S*. *maltophilia* (BurA1, BurE1, PierC1, R551.3, MEEB-Am6.2) were determined to be non-virulent strains as fewer than 400 amoebae were needed to form lysis plaques such as *K*. *pneumoniae* strain. Two strains of *S*. *maltophilia* (MEEB-Am6.1, K279A) were characterized as virulent with a similar effect as the control strain *P*. *aeruginosa* PT5. None of the *S*. *maltophilia* strains tested presented a low-virulent phenotype.

### Internalization, intracellular growth and virulence of *S*. *maltophilia* BurA1Δ*eby*CAB

In order to evaluate the role of efflux pumps in the survival and multiplication of environmental *S*. *maltophilia* isolates inside amoeba, we chose the model *BurA1* and its mutant BurA1Δ*eby*CAB which lacks the *eby*CAB efflux pump gene previously described.

**Fig 6:**
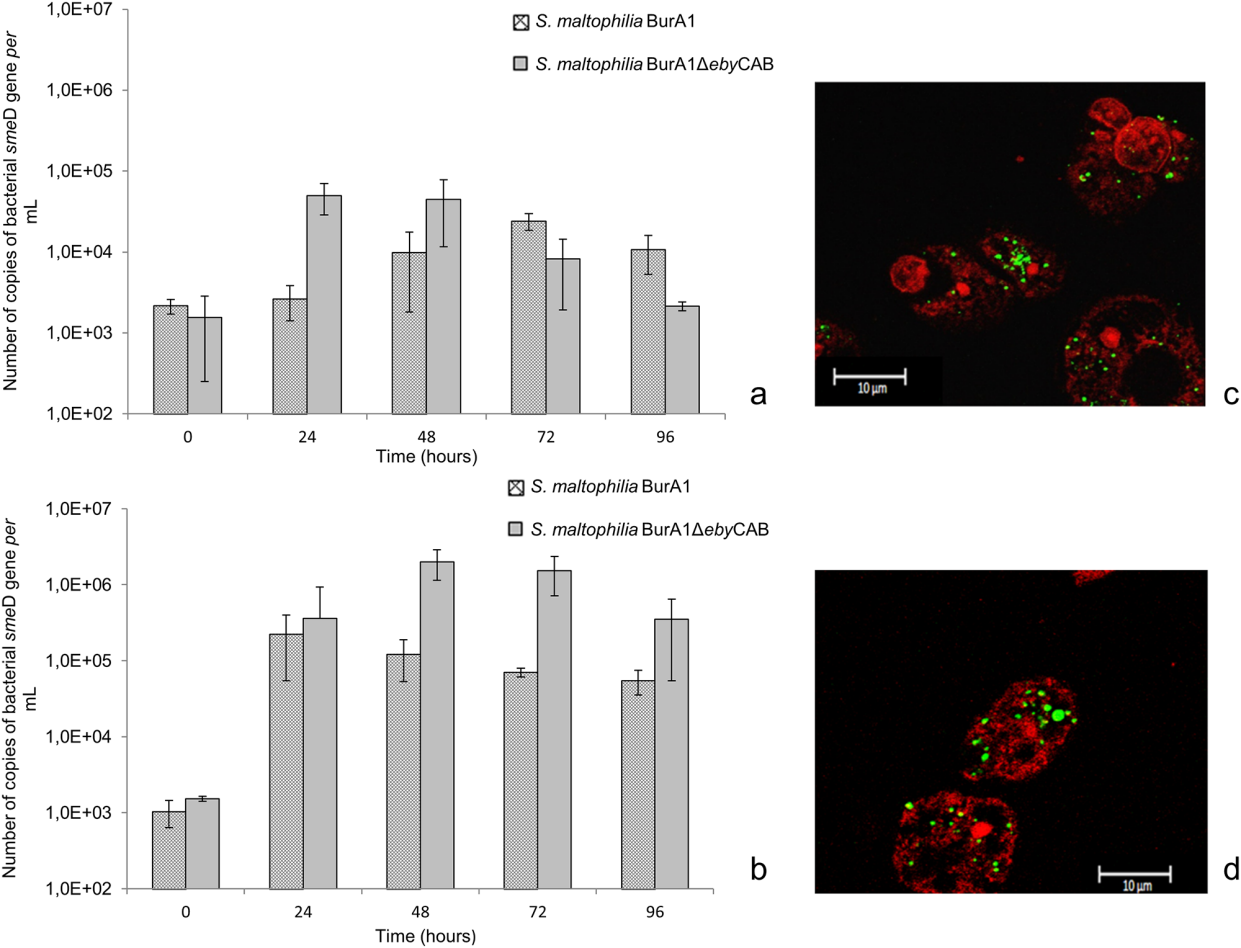
Growth of *S*. *maltophilia* BurA1 and BurA1Δ*eby*CAB expressed in number of copies of bacterial *sme*D gene *per* mL, in co-culture with amoeba and confocal microscopy. (a) co-culture *with A*. *castellanii* L6a; (b) co-culture with *W*. *magna* C2c. Means +/- standard deviations from three independent experiments in duplicate are presented; (c) Fluorescent confocal microscopy images of *A*. *castellanii* in co-culture with *S*. *maltophilia* BurA1Δ*eby*CAB after 48 hours; (d) Fluorescent confocal microscopy images of *W*. *magna* in co-culture with *S*. *maltophilia* BurA1Δ*eby*CAB after 48 hours.

At the beginning of the co-culture experiments (0 h), with both amoebae, the number of internalized *S*. *maltophilia* BurA1 and BurA1Δ*eby*CAB was about 1.5 x 10^3^ cells *per m*l. In co-culture with both species of amoebae, *S*. *maltophilia* BurA1Δ*eby*CAB was able to survive and multiply inside amoebae (Figs 6a and 5b). With *A*. *castellanii*, the number of *S*. *maltophilia* BurA1Δ*eby*CAB at 24 hours was multiplied by 32 compared to time 0. With *W*. *magna*, the population of *S*. *maltophilia* BurA1Δ*eby*CAB at 48 hours was multiplied by a factor of 1307 compared to time 0. Confocal microscopy confirmed the presence of BurA1Δ*eby*CAB cells inside both amoebae (Fig 6c and 6d).

At the end of the experiment with *A*. *castellanii* L6a, the number of *S*. *maltophilia* BurA1Δ*eby*CAB cells was lower than with the wild type strain. However, with *W*. *magna*, the number of *S*. *maltophilia* BurA1Δ*eby*CAB cells was higher than with *S*. *maltophilia* BurA1.

**Fig 7:**
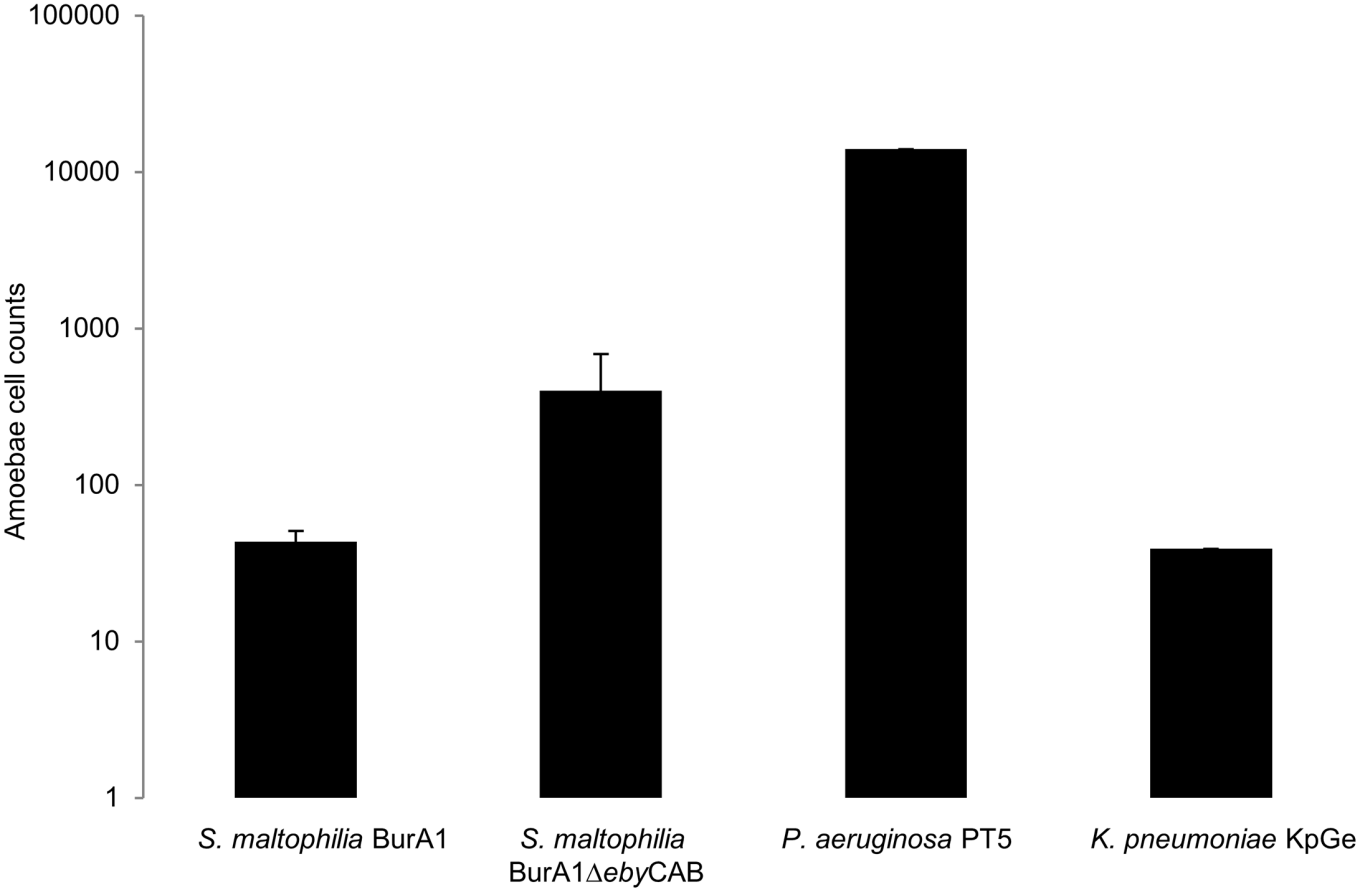
*D*. *discoideum* plate killing assay with two *S*. *maltophilia* strains, BurA1 and BurA1Δ*eby*CAB. Bars representing the number of amoebae necessary to form a lysis plaque on the bacterial lawn. *Pseudomonas aeruginosa* PT5 and *Klebsiella pneumoniae* KpGe are used as negative and positive controls, respectively. Means +/- standard deviations from three independent experiments in triplicate are presented.

Regarding virulence assays, Fig 7 showed that *S*. *maltophilia* BurA1Δ*eby*CAB was considered to be a low virulence strain because 399 cells of *D*. *discoideum* were necessary to form a lysis plaque, whereas the wild type strain was non-virulent because only 43 cells of *D*. *discoideum* were necessary to form a lysis plaque.

## Discussion

Free-living amoebae may constitute a host for some bacterial species [33]. Currently, most studies have focused on species known to be endosymbionts of *Acanthamoeba*, such as the members of the bacterial genera *Legionella*, *Chlamydia* or *Mycobacterium avium* [34]. Other analyses characterizing amoebal microbiomes have shown the presence of various associated bacteria including several human opportunistic pathogens, such as *P*. *aeruginosa* and *S*. *maltophilia* [17,35,36]. The present study demonstrated that various strains of *S*. *maltophilia* regardless of their origin i.e. environmental or clinical, were capable of intra-cellular survival and/or growth inside two different FLA, *A*. *castellanii* and *W*. *magna*. Recently, Cateau *et al*. (2014) [26] also reported that both clinical and environmental isolates of *S*. *maltophilia* were able to survive and multiply inside *V*. *vermiformis*. Our results are the first to compare the behavior of several environmental isolates of *S*. *maltophilia* with two amoebal genera and provide insight on whether selectivity towards specific amoebal genera exists or not. We showed that at an early step of the interaction i.e. bacterial internalization (time 0) by the amoeba, differences can be seen in the number of cells internalized from one strain to another. Indeed, with *A*. *castellanii* L6a, the numbers of *S*. *maltophilia* PierC1 and R551.3 cells internalized is lower than for the other strains. Regarding W. *magna* C2c, *S*. *maltophilia* PierC1 and K29a were the least internalized strains. These differences were confirmed using culture approach.

This observation could be related to differences in the affinity of the amoeba towards *S*. *maltophilia* strains or to differences in bacterial strategies to escape phagocytosis [37].

Also, the ability of *S*. *maltophilia* to grow inside amoeba is different according to bacterial strains and amoebal genera. For instance, strains BurA1 and BurE1 were able to grow inside *A*. *castellanii* L6a, whereas others such as K279a showed the highest growth rate within *W*. *magna* C2c. Furthermore, *S*. *maltophilia* K279a was found unable to multiply inside *Acanthamoeba* but increased by 3 log in *W*. *magna*. These results clearly showed that the ability of bacteria to survive or multiply in amoeba varies between bacterial strains and that a strain might grow very well in one amoebal species and not in the other. Corsaro *et al*. (2013) demonstrated that a *S*. *maltophilia* strain was able to multiply inside *A*. *castellanii* but not in *Naegleria lovaniensis* [27]. The variability of bacterial proliferation was also reported in a previous study where the multiplication of *Legionella pneumophilia* differed inside *Acanthamoeba*, *Hartmanella* and *Willaertia* [24]. Interestingly, Dey *et al*. (2009) [24] showed that *W*. *magna* C2c was very resistant towards the proliferation of *L*. *pneumophila* Paris but not towards other strains of *L*. *pneumophila*. Our study showed that *W*. *magna* C2c was more permissive to *S*. *maltophilia* strains than *A*. *castellanii* L6a.

We noted that after 96 hours of co-culture experiments, no lysis of both amoebae was observed regardless of the strain of *S*. *maltophilia* used and no bacterial cells were present in the co-culture medium. These data agree with the partial lysis observed only after 5 day of co-culture between *S*. *maltophilia* and *V*. *vermiformis* in the study of Evstigneeva *et al*. (2009) [38]. The incubation period of our co-culture experiment could be increased by a few days in order to determine if *S*. *maltophilia* would be able to lyse amoeba and to persist in the environment like the species *L*. *pneumophila* [39].

Our study involved two approaches to evaluate bacterial survival in amoebae. The use of qPCR approach was coupled to culture approach and provided data on the viability of *S*. *maltophilia* strains inside amoebae. We showed that the qPCR approach and the culture one led to the same trend for *S*. *maltophilia* multiplication. To our knowledge our study is the first one that combines both approaches to study bacteria-amoebae interactions as previous reports were based on one or the other approach [24,26,27,40].

In order to survive or multiply inside amoebae, ARB could express virulence genes, such as those reported for *L*. *pneumophila* [40] and *Chlamydia* spp. which possess the type III secretion system as an important virulence feature for adherence and host cell invasion [41]. We used the social amoebae *D*. *discoideum* and showed that two out of seven strains of *S*. *maltophilia*, MEEB-Am6.1 and K279a, could be considered as virulent strains. This finding is interesting as BurA1 and BurE1 were able to multiply in amoebae and were not virulent, while S. *maltophilia* MEEB-Am6.1 was virulent but unable to survive and grow in both amoebal species. A similar observation was previously reported from a collection of 59 isolates of *S*. *maltophilia* tested in interaction with *D*. *discoideum* and *A*. *castellanii* [23]. One can hypothesize that the virulence factors of *S*. *maltophilia* strains involved in the interaction with *D*. *discoideum* might be different from those involved in the interaction with *A*. *castellanii* or *W*. *magna*. However, it is important to note that our virulence tests were performed at 22.5°C, whereas our co-culture experiments were performed at 32°C. Temperature could be partly responsible for these differences as some bacterial strains express their virulence traits at temperatures higher than 22.5 °C [33].

The genome of *S*. *maltophilia* was shown to harbor numerous efflux pumps and these pumps are increasingly recognized as having a role in bacterial physiology and virulence [42]. For instance, the AcrAB-TolC pump of *Enterobacter cloacae* was found to be involved in virulence [43]. We thus investigated the role of EbyCAB efflux pump previously described [7] and showed that in both amoebae, *S*. *maltophilia* BurA1Δ*eby*CAB multiplied more than the wild type strain and exhibited a higher virulence than the wild type strain. Whether the efflux pump EbyCAB is involved in virulence is still unclear but the expression of this pump seems to decrease the fitness of *S*. *maltophilia* BurA1. The exact role of this pump needs to be investigate further as the overexpression of a MDR pump in *S*. *maltophilia* was found related to a decrease of fitness and a lower virulence [44].

## Conclusion

In conclusion, our results showed for the first time that *S*. *maltophilia* isolates with contrasting phenotypes of virulence are able to grow inside two amoebae, *A*. *castellanii* and *W*. *magna*. These results suggest that in the environment, *S*. *maltophilia* could have the potential to infect and proliferate within a large panel of FLA. The fact that this emerging opportunistic pathogen is often found in the amoebal microbiome [35] and that it can multiply in the amoeba support the hypothesis that in the environment, FLA could be a reservoir and vector for the transmission of *S*. *maltophilia*. Thus, *S*. *maltophilia* could use the amoebae as a “training ground” in order to better resist human macrophages, as demonstrated for *L*. *pneumophila* [39], or to increase their virulence and antibiotic resistance properties [45]. In conclusion, FLA constitute an ecological niche for opportunistic bacterial pathogens in which important genetic exchanges between species could occur and contribute to the propagation of antibiotic resistance and virulence genes in the environment [46].

## Supporting information

S1 Fig Genetic organization surrounding *eby*CAB operon of *S*. *maltophilia* BurA1 and structure of the recombinant plasmid used in this study (a), primers used to construct the *eby*CAB isogenic mutant of *S*. *maltophilia* BurA1 (b). The gene orientation is indicated by arrows. White box: deleted region. Underlined nucleotides represent the nucleotides added to create a complementary region between upstream and downstream fragments.
(TIF)
