# Supplementary figures and images for: Survival and growth of Stenotrophomonas maltophilia in free-living amoebae (FLA) and bacterial virulence properties
Source: PLoS One. 2018 Feb 5;13(2):e0192308. doi: 10.1371/journal.pone.0192308 (PMC5798789; doi:10.1371/journal.pone.0192308)

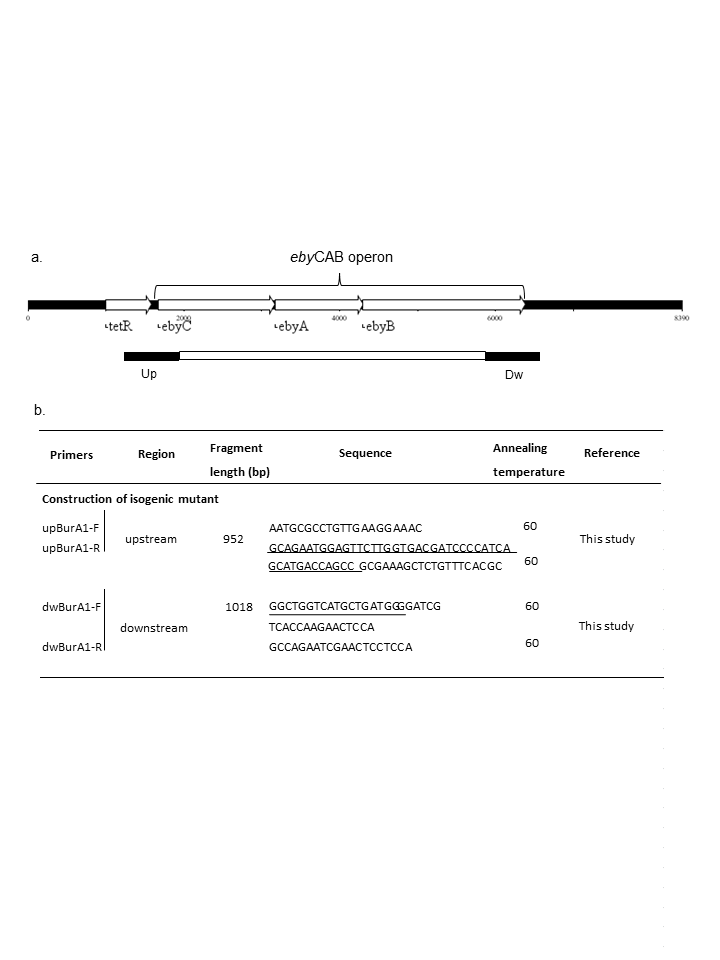

Supplement: S1 Fig — The gene orientation is indicated by arrows. White box: deleted region. Underlined nucleotides represent the nucleotides added to create a complementary region between upstream and downstream fragments. (TIF) [file pone.0192308.s001.tif]
